# Review Article: Renal Safety Profiles of Tenofovir Alafenamide, Tenofovir Disoproxil Fumarate, and Entecavir for the Treatment of Chronic Hepatitis B Infection—General and Special Populations

**DOI:** 10.1111/apt.70560

**Published:** 2026-02-03

**Authors:** Lung‐Yi Mak, Tsung‐Hui Hu, Desmond Y. H. Yap

**Affiliations:** ^1^ Division of Hepatology and Gastroenterology, Department of Medicine Queen Mary Hospital, The University of Hong Kong Pok Fu Lam Hong Kong; ^2^ Division of Hepato‐Gastroenterology, Department of Internal Medicine Kaohsiung Chang Gung Memorial Hospital and Chang Gung College of Medicine Kaohsiung Taiwan; ^3^ Division of Nephrology, Department of Medicine Queen Mary Hospital, the University of Hong Kong Pok Fu Lam Hong Kong

**Keywords:** chronic hepatitis B, entecavir, nephrotoxicity, tenofovir

## Abstract

**Background:**

Renal safety is an important consideration for treatment selection in chronic hepatitis B (CHB) because of the ageing population and increasing prevalence of medical comorbidities. However, the renal safety profiles of first‐line nucleos(t)ide analogues (NUCs) for CHB—tenofovir alafenamide (TAF), tenofovir disoproxil fumarate (TDF), and entecavir (ETV) have not been comprehensively reviewed.

**Aims:**

To evaluate the renal safety of ETV, TDF, and TAF in general and special populations with CHB.

**Methods:**

In this narrative review, relevant studies in PubMed were identified using a range of keywords, followed by manual screening of reference lists to capture additional sources.

**Results:**

Based on current randomised and real‐world evidence, TDF may cause more nephrotoxic effects than ETV in patients with pre‐existing moderate‐to‐severe chronic kidney disease (CKD), but the two agents may have similar renal safety profiles among patients with no or mild baseline renal impairment. Randomised data showed that TAF is significantly less nephrotoxic than TDF in different clinical settings. Retrospective data from both treatment‐naïve and ‐experienced patients, as well as special populations, including patients with renal impairment, kidney transplant, advanced age, and acute‐on‐chronic liver failure, indicated that TAF may be more likely to improve renal function compared to ETV.

**Conclusions:**

Current first‐line NUCs show comparable renal safety profiles in CHB patients with no or mild kidney dysfunction, with growing evidence that favours TAF. Future prospective studies are needed to validate these findings, and more research should focus on CHB patients with diabetes mellitus who are at risk of CKD.

## Graphical abstract

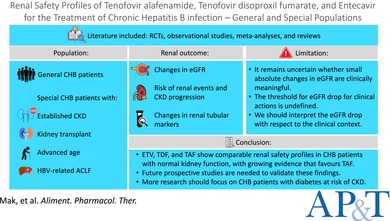

**Abbreviations**
ACLF: acute‐on‐chronic liver failure
CHB: chronic hepatitis B
CI: confidence interval
CKD: chronic kidney disease
DM: diabetes mellitus
eGFR: estimated glomerular filtration rate
ESRD: end‐stage renal disease
ETV: entecavir
GFR: glomerular filtration rate
HBV: hepatitis B virus
HR: hazard ratio
IQR: interquartile range
IRR: incidence rate ratio
KTR: kidney transplant recipient
NUC: nucleos(t)ide analogue
OAT: organic anion transporter
OR: odds ratio
PSM: propensity score‐matched
RCT: randomised controlled trial
SMD: standardised mean difference
SUCRA: surface under the cumulative ranking curve
TAF: tenofovir alafenamide
TDF: tenofovir disoproxil fumarate

## 1 Introduction

Hepatitis B virus (HBV) infection poses a substantial global disease burden, affecting approximately 296 million individuals and representing a leading cause of liver cirrhosis and hepatocellular carcinoma, contributing significantly to mortality worldwide [1]. In addition to the liver, HBV infection impacts the kidneys, showing associations with diseases such as membranous nephropathy, membranoproliferative glomerulonephritis, and polyarteritis nodosa [2]. Up to 65% of antiviral‐naïve patients with HBV infection have renal conditions, including proteinuria, haematuria, glycosuria, and non‐infectious leukocyturia [3]. Furthermore, meta‐analyses have revealed that HBV infection is a significant risk factor for chronic kidney disease (CKD) and end‐stage renal disease (ESRD) [4, 5]. In the context of population ageing, renal impairment is becoming an increasingly pressing concern among patients with chronic hepatitis B (CHB). Notably, even healthy individuals experience an average decline of 8 mL/min in glomerular filtration rate (GFR) for every decade after the age of 40 years [6]. Data from both Western and Asian populations show that ageing with CHB is associated with increasing comorbidities, including CKD, diabetes mellitus (DM), and hypertension (HT) [7, 8]. DM and HT are important risk factors for the development of CKD in Chinese patients with CHB [9]. One study from Hong Kong reported that patients with type 2 DM and CHB are associated with significantly increased risk of ESRD (adjusted hazard ratio [HR], 4.53; 95% confidence interval [CI], 1.11–18.58; *p* = 0.036) [10].

Given the absence of curative treatment [11], most patients with CHB require lifelong antiviral therapy with a nucleos(t)ide analogue (NUC). Although antiviral therapy can lower the risk of kidney disease in CHB [12], NUCs differ in renal safety profiles, making potential long‐term nephrotoxicity an important consideration for treatment selection. Guideline‐recommended first‐line NUC therapies for HBV infection include tenofovir alafenamide (TAF), tenofovir disoproxil fumarate (TDF), and entecavir (ETV) [13, 14, 15]. Notably, TDF is generally considered to have nephrotoxic potential, where a switch to TAF or ETV is recommended if patients on TDF experience kidney or bone disorder (Table 1) [13, 14, 15]. This review focuses on the renal safety profiles of first‐line NUCs for CHB in both general and special populations.

**TABLE 1:** Guideline‐recommended conditions that require avoidance of tenofovir disoproxil fumarate (TDF) treatment in patients with chronic hepatitis B.

Guideline	EASL 2025 [13]	WHO 2024 [14]	AASLD/IDSA 2025 [15]
	– GFR decreases; – Tubulopathy occurs; or – Hypophosphatemia or osteoporosis develops	– CrCl falls below 50 mL/min; – Progressive decline of renal function occurs; or – Low bone mineral density is detected or suspected because of a fracture	– Presence of renal or bone disease

Abbreviations: AASLD, American Association for the Study of Liver Diseases; CrCl, creatinine clearance; EASL, European Association for the Study of the Liver; GFR, glomerular filtration rate; IDSA, Infectious Diseases Society of America; WHO, World Health Organization.

## 2 Methods

This narrative review involved searching the PubMed database for the publication period between October 2015 and October 2025, using the following keywords: ‘entecavir’, ‘tenofovir’, ‘renal function’, ‘kidney’, ‘glomerular filtration rate’, ‘nephrotoxicity’, ‘kidney transplant’, ‘diabetes’, ‘elderly’ and ‘older’. The language of the literature was English or Chinese. The titles and abstracts from search results were screened to identify target articles that included randomised controlled trials (RCTs), post hoc analyses, observational studies, meta‐analyses, systematic reviews, and narrative reviews addressing renal outcomes of ETV, TDF, and TAF (e.g., changes in estimated GFR [eGFR], CKD stage, urinary biomarkers) among treatment‐naïve or treatment‐experienced patients with CHB, including those with advanced age or other underlying conditions (e.g., established CKD, kidney transplant, liver failure). References in identified articles were also manually screened for additional relevant sources.

## 3 Renal Safety Profile of ETV vs. TDF

**FIGURE 1:**
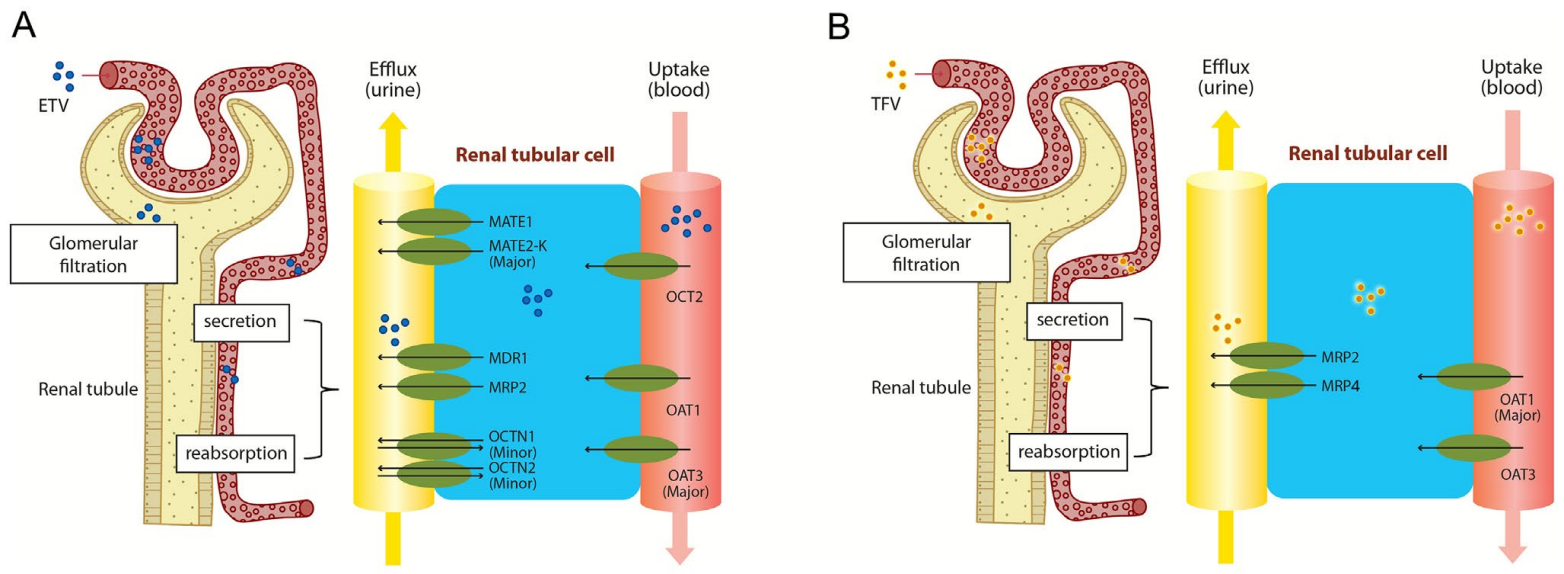
Drug transporters of entecavir (ETV; A) and tenofovir (TFV; B) that are involved in renal efflux and renal uptake [23]. MATE, multi‐antimicrobial extrusion protein; MDR, multidrug resistance protein; MRP, multidrug resistance‐associated protein; OAT, organic anion transporter; OCT, organic cation transporter; OCTN, organic cation/carnitine transporter.

Acute and chronic nephrotoxicity is an important concern for using tenofovir (TFV)‐related compounds [16]. The entry of TFV into proximal renal tubular cells is mediated through the organic anion transporters (OAT1 and OAT3), and its intracellular accumulation will cause mitochondrial damage with morphological (enlargement) and functional changes [17]. Conventional views consider ETV to be more kidney‐friendly than TDF [13, 14, 15], but recent clinical studies have revealed some mixed findings [18, 19, 20, 21, 22]. From a pharmacological perspective, the metabolism of both ETV and TDF is markedly kidney‐dependent [17, 23]. The renal uptake of ETV is likely mediated by OAT1/3 and organic cation transporter 2, with multiple other drug transporters involved in both renal secretion and reabsorption (Figure 1) [23].

### 3.1 Changes in eGFR

An RCT of 400 nucleoside‐naïve patients with CHB in Thailand showed that subjects treated with ETV or TDF (randomised 1:1) experienced a similar rate of drop in mean eGFR from baseline through weeks 48, 96, and 114 [18]. The proportion of patients having eGFR decline ≥ 20% at week 144 (the primary endpoint) did not differ between the treatment groups (ETV, 14.9% vs. TDF, 16.8%; *p* = 0.628) [18].

Multiple real‐world studies demonstrated that, after adjusting for baseline renal function or restricting analyses to patients without pre‐existing renal impairment, there was no significant difference in nephrotoxicity between ETV and TDF [19, 20, 21, 22]. A 1:1 retrospective propensity score‐matched (PSM) study in the United States revealed that, among patients with baseline eGFR ≥ 60 mL/min/1.73 m^2^, those treated with ETV or TDF (*n* = 116 each) had similar mean eGFRs during a follow‐up of 43–46 months [24]. Cox regression analysis also showed that TDF was not associated with renal impairment compared with ETV [24]. A lower mean eGFR (44.7 vs. 50.8 mL/min/1.73 m^2^; *p* < 0.0001) with TDF (*n* = 26) versus ETV (*n* = 32) was only observed in unmatched data from the patient subgroup with eGFR < 60 mL/min/1.73 m^2^ [24].

A recent systematic review and meta‐analysis of real‐world studies concluded that ETV and TDF had comparable effects on serum creatinine levels at 12 months, 18–24 months, and > 24 months of treatment [25]. Additionally, TDF was associated with greater reductions in eGFRs compared with ETV at 12 months (standardised mean difference [SMD], −0.07; 95% CI, −0.12 to −0.01; *p* = 0.02) and 18–24 months (SMD, −0.11; 95% CI, −0.17 to −0.05; *p* = 0.0002) [25]. However, these differences were numerically small, and both agents led to similar reductions in eGFRs after 24 months (SMD, −0.03; 95% CI, −0.22 to 0.16; *p* = 0.77) [25].

### 3.2 Risk of Renal Events and CKD Progression

A retrospective PSM study in Taiwan showed that TDF (*n* = 253) was associated with a higher risk of renal dysfunction (HR, 5.36; 95% CI, 2.16–13.35; *p* < 0.001) compared with ETV (*n* = 506) over 48 months; however, pre‐existing CKD (HR, 6.71; 95% CI, 2.25–17.65), proteinuria (HR, 3.39; 95% CI, 1.23–9.39), and haematuria (HR, 4.25; 95% CI, 1.32–13.68) were also identified as risk factors for renal dysfunction [26].

An analysis of medical records from several European countries found that TDF was associated with more renal events than ETV in CHB patients with moderate‐to‐severe renal impairment, either pre‐ or post‐antiviral treatment initiation [27].

While a recent retrospective study showed that TDF was associated with worse renal outcomes compared with ETV during a 10‐year follow‐up [28], further prospective studies are warranted to validate these findings and assess their generalisability.

### 3.3 Summary of Evidence

Despite limitations such as selection bias and heterogeneities in follow‐up duration and patient characteristics (e.g., age and comorbidities), real‐world evidence, supplemented by one RCT, indicates that the comparative nephrotoxicity of ETV and TDF remains uncertain, especially among patients without baseline renal impairment.

## 4 Renal Safety Profile of TAF vs. TDF

While both TAF and TDF are prodrugs of TFV, there is robust evidence to support a significantly improved renal safety profile for TAF when compared to TDF [29, 30, 31]. In this regard, TAF exhibits better plasma stability, where plasma TFV levels derived from standard‐dose TAF (25 mg) are approximately 92% lower than that from normal‐dose TDF (300 mg) [32, 33]. In plasma, TDF is rapidly hydrolysed to TFV, which is renally eliminated by glomerular filtration and active secretion via OATs (Figure 1), whereas TAF is not a substrate of renal OATs and thus has significantly lower drug accumulation within renal cells [17, 34].

### 4.1 Changes in eGFR

In long‐term RCTs, patients on TAF for 8 years showed minimal changes in eGFR, whereas patients on TDF for 2–3 years had a decrease in eGFR, and such changes improved after switching to TAF (Table 2) [29]. Consistent results were also observed in the East Asian patient subgroup from these studies [30]. One RCT in patients who achieved HBV suppression with TDF for ≥ 48 weeks demonstrated that a switch from TDF to TAF significantly improved eGFR at week 48 (median change, 0.94 vs. −2.74 mL/min; *p* < 0.0001) compared with continuation of TDF [31].

**TABLE 2:** Changes in renal outcomes from baseline to year 8 among patients of phase 3 randomised Studies 108 and 110 [29].

Treatment arm	TAF8y (*n* = 775)	TDF2y→TAF6y (*n* = 180)	TDF3y→TAF5y (*n* = 202)
CKD stage change	Stage 1→Stage 1: 84% Stage 2→Stage 1: 62%	Stage 1→Stage 1: 84% Stage 2→Stage 1: 65%	Stage 1→Stage 1: 77% Stage 2→Stage 1: 67%
Median change in eGFR_CG_, mL/min	−5.4	−5.0	−4.9
Median percent change in B_2_M:Cr, %	+11.0	+8.8	+29.7
Median percent change in RBP:Cr, %	+43.2	+53.0	+49.8

Abbreviations: B_2_M:Cr, ratio of beta 2‐microglobulin to creatinine; CKD, chronic kidney disease; eGFR_CG_, estimated glomerular filtration rate by Cockcroft‐Gault method; RBP:Cr, ratio of retinol‐binding protein to creatinine; TAF8y, double‐blind tenofovir alafenamide treatment for 8 years; TDF2y→TAF6y or TDF3y→TAF5y, double‐blind tenofovir disoproxil fumarate treatment for 2 or 3 years followed by open‐label tenofovir alafenamide treatment for 6 or 5 years.

### 4.2 Risk of Renal Events and CKD Progression

Pooled analyses of two RCTs indicated that 84% of patients remained in stage 1 CKD when they were treated with upfront TAF for 8 years (Table 2) [29]. For patients who switched from TDF to TAF at 2 or 3 years, the rates of downstaging from stage 2 CKD to stage 1 CKD were 65% and 67%, respectively (Table 2) [29], implying that an earlier switch from TDF to TAF might confer more renal benefits.

### 4.3 Changes in Renal Tubular Markers

The pooled data from RCTs demonstrated that TAF therapy was associated with little changes in markers of proximal tubulopathy, and in contrast TDF treatment for 2–3 years caused an increase in markers of tubular dysfunction, and such changes improved after conversion to TAF (Table 2) [29].

## 5 Renal Safety Profile of TAF vs. ETV

Despite its improved nephrotoxicity profile when compared to TDF, whether TAF shows similar or better renal safety than ETV remains unclear. The current guidelines for CHB management suggest that TAF and ETV have comparable renal safety profiles [13, 14, 15]. However, as TAF is a relatively new standard‐of‐care NUC for CHB, it is also worthwhile to evaluate its nephrotoxicity relative to ETV in the light of emerging real‐world and long‐term studies.

### 5.1 Changes in eGFR

Retrospective data from 167 treatment‐naïve Chinese patients who received ETV (*n* = 117) or TAF (*n* = 50) for ≥ 48 weeks showed that, after 2:1 PSM, the ETV group (*n* = 100) showed a significantly lower eGFR (106.42 ± 14.12 vs. 112.25 ± 13.44 mL/min/1.73 m^2^; *t* = −2.422; *p* = 0.017) and a significantly higher incidence of abnormal renal function (17.00% vs. 4.00%; *χ* ^2^ = 5.092; *p* = 0.024) compared with the TAF group (*n* = 50) at week 48 [35]. Multivariate regression analysis identified ETV (odds ratio [OR] = 5.589; 95% CI, 1.136–27.492; *p* = 0.034) and baseline eGFR (OR = 0.896; 95% CI, 0.841–0.955; *p* < 0.001) as independent risk factors for abnormal renal function [35].

Li et al. reported that, among patients with baseline eGFR ≥ 90 mL/min/1.73 m^2^, treatment initiation with ETV (*n* = 252) or TAF (*n* = 143) did not significantly affect the rate of developing CKD (13.9% vs. 9.8%; *p* = 0.304) during a follow‐up of ~20 months [36]. However, the TAF group showed a significantly lower overall rate of eGFR decline every 12 weeks compared with the ETV group (adjusted difference, 0.38 mL/min/1.73 m^2^; 95% CI, 0.11–0.65; *p* = 0.006) [36].

Multiple real‐world studies demonstrated that a switch from ETV to TAF may improve renal function in patients with CHB. In a study of relatively young patients (median age, 34–35 years) from China, PSM data on 84 pairs showed that those treated with ETV had a significantly lower median eGFR (110.0 vs. 115.9 mL/min; *p* = 0.015) compared with those treated with TAF at week 48 [37]. Additionally, six patients were converted from ETV to TAF after their eGFRs dropped to below 90 mL/min (range, 60–89 mL/min), and such substitution resulted in significant renal function improvements from week 48 to 60 (eGFRs: 83.60 mL/min at week 48 to 93.39 mL/min at week 60; *p* = 0.031) [37]. However, no statistically significant changes in serum creatinine levels or eGFRs were observed from weeks 60 to 72, implying that an early switch to TAF may benefit ETV‐treated patients with stage 2 CKD by preventing further renal deterioration [37]. A prospective observational analysis of 77 HBV‐suppressed Japanese patients assigned to continue ETV (*n* = 31) or switch to TAF (*n* = 46) showed that, despite a lack of statistical significance, continuing ETV was associated with a numerically larger decline in eGFR compared with switching to TAF (−5.4 vs. −2.7 mL/min/1.73 m^2^; *p* = 0.240) at week 240, with no significant between‐group differences in other urinary biomarkers [38].

In a network meta‐analysis of 16 real‐world studies that included a total of 4278 patients treated with ETV, TAF, or TDF for CHB, TAF appeared to confer more beneficial effects on creatinine levels compared with ETV (SMD = −0.55; 95% CI, −0.09 to −1.01; *p* value not reported) [39]. While there were no significant differences in the effect of these agents on eGFR (SMD = −0.67; 95% CI, −2.10 to 0.75; *p* value not reported), the surface under the cumulative ranking curve (SUCRA) demonstrated that TAF had a substantially lower probability of eGFR reduction than ETV (8.8% vs. 41.2%) [39]. The eGFR outcomes should be interpreted cautiously because of the limited sample size [39].

### 5.2 Risk of Renal Events and CKD Progression

In a retrospective study of 1988 treatment‐naïve patients who received ETV (*n* = 1839) or TAF (*n* = 149) in South Korea, each treatment group included 149 patients after PSM [40]. The ETV group had a significantly higher risk of progression in CKD stage ≥ 1 compared with the TAF group (incidence, 19.9 vs. 5.1 per 1000 person‐years; adjusted HR, 4.05; 95% CI, 2.14–7.68; *p* < 0.001) [40].

Another retrospective study from South Korea included 1061 untreated patients with CHB, 366 patients on TAF, and 2029 on ETV [41]. Compared with each 1:1 PSM untreated group, the ETV group (*n* = 541) had a significantly higher risk of renal function decline (defined as a minimum one‐stage increase in CKD for ≥ 3 consecutive months; adjusted HR, 1.53; 95% CI, 1.33–1.75; *p* < 0.001), in contrast to no significant difference in this outcome for the TAF group (*n* = 222; adjusted HR, 1.89; 95% CI, 0.87–4.10; *p* = 0.107) [41].

In a large study of 10,642 patients with CHB, compared with respective PSM untreated cohorts, those treated with ETV (*n* = 755) or TAF (*n* = 426) had no increased risk of CKD progression (i.e., a minimum one‐stage elevation), while PSM data (510 pairs) showed that TAF was associated with a numerically lower incidence of CKD progression compared with ETV (5.2 vs. 6.0 per 100 person‐years; *p* = 0.118) [42].

In a recent analysis of the nationwide health insurance database from South Korea, pre‐PSM data on treatment‐naïve patients with CHB indicated that, compared with TAF, ETV was associated with a significantly higher CKD incidence (incidence rate ratio [IRR], 2.43; 95% CI, 2.13–2.77; *p* < 0.001) in those with normal renal function at baseline, and a significantly higher ESRD incidence (IRR, 1.82; 95% CI, 1.26–2.63; *p* = 0.001) in those with baseline CKD [43]. PSM data showed that these incidences were numerically higher with ETV than TAF (CKD IRR, 1.20; 95% CI, 0.69–2.10; *p* = 0.523; ESRD IRR, 1.49; 95% CI, 0.81–2.75; *p* = 0.198). However, Cox regression analysis did not pinpoint any NUC as a significant risk factor for CKD or ESRD [43].

In a multinational study of 425 patients (~90% Asian) who received ETV for an average of 6 years, switching to TAF did not significantly affect CKD progression at week 96 [44]. However, during follow‐up, downstaging of CKD were more frequent than upstaging after switching to TAF: 18% (27/151) of patients improved from stage 2 to 1, and 19% (7/37) from stages 3–5 to 2, compared with 11% (26/235) who progressed from stage 1 to 2 and 8% (12/151) from stage 2 to stages 3–5 [44].

### 5.3 Changes in Renal Tubular Markers

Apart from affecting filtration function (i.e., GFR or CrCl), NUCs can also cause proximal renal tubular dysfunction– often characterised by impaired phosphate reabsorption [45]. Abnormal phosphate handling in the kidneys can lead to defective bone mineralisation, osteomalacia, and increased risk of fractures [45].

In a network meta‐analysis, TAF and ETV did not significantly differ in the effects on bone mineral density (BMD) or blood phosphorus [39]. Compared with ETV, TAF demonstrated a lower probability of reducing BMD (SUCRA, 19.6% vs. 50.6%) but a higher probability of reducing blood phosphorus (SUCRA, 49.8% vs. 9.7%) [39]. These findings were inconclusive because only a few relevant studies on TAF were included in the analysis [39].

A recent meta‐analysis that focused on comparing ETV and TAF concluded that TAF was associated with a more favourable overall safety profile; however, further studies are warranted to assess differences in specific adverse events (e.g., renal function decline) between these two agents [46].

### 5.4 Summary of Evidence

Retrospective data suggest a trend toward improved renal function for TAF over ETV. In the real‐world setting, physicians' choice of NUC created inherent bias in patient characteristics, where patients deemed at‐risk of renal impairment would be prescribed ETV rather than TFV‐containing NUCs. The reimbursement policy for NUCs is another potential source of unadjusted confounding when patients with pre‐existing medical comorbidities are less likely to receive TAF due to drug cost considerations.

Although absolute changes in eGFR appear to be numerically minimal among patients treated with TAF or ETV, one should appreciate that the studies were relatively short‐term and that the small or non‐significant differences in eGFRs may translate into clinically important effects on kidney function with a prolonged exposure to these NUCs, especially among patients with advanced age or comorbidities (e.g., CKD, DM, HT) who are expected to experience a faster decline in eGFR [47]. In the general population, the expected rate of eGFR decline is ~0.9 mL/min/1.73 m^2^ per year, and a 15%–20% annual reduction in eGFR represents substantial functional deterioration [47, 48]. Long‐term RCTs using these thresholds of renal impairment progression are warranted to determine whether TAF and ETV lead to a clinically significant impact on kidney function and the genuine difference in their renal safety profiles.

## 6 Renal Safety of ETV vs. TAF in Special Populations

The changing patient demographics such as population ageing and growing prevalence of medical comorbidities have posed new challenges for the management of CHB. ETV and TAF are widely considered to be preferred over TDF in vulnerable individuals (e.g., the elderly and patients with renal or bone disease) [13, 14, 15]. This section reviews the comparative renal safety profiles of ETV and TAF in CHB populations at risk of or with established renal dysfunction.

### 6.1 Patients With Established CKD

A phase 1 study evaluated the pharmacokinetics of TAF and its active form, TFV, in HBV‐uninfected individuals with severe renal impairment (eGFR, 15–29 mL/min; *n* = 14) and matched healthy controls (eGFR ≥ 90 mL/min; *n* = 13) [49]. Although the plasma exposures of TFV from TAF 25 mg were higher in individuals with severe renal impairment than in controls with normal renal function, they were nonetheless lower than the historical TFV exposures from TDF 300 mg in individuals with normal renal function, highlighting the superior renal safety of TAF over TDF [49]. Additionally, TAF mainly undergoes hepatobiliary clearance, and hence renal impairment will incur little pharmacokinetic changes on drug metabolism [49].

An open‐label multicentre phase 2 study investigated the effect of switching to TAF in HBV‐suppressed patients with renal (Part A) or hepatic impairment (Part B) [50]. Part A included two cohorts: (1) 78 patients with moderate or severe renal impairment (median eGFR, 45.7 mL/min); and (2) 15 patients with ESRD on haemodialysis (median eGFR, 7.3 mL/min) [50]. At baseline, 57/93 (61%) and 28/93 (30%) received TDF (median duration of use, ~5.5 years) and ETV (median duration of use, ~4.6 years), respectively [50]. In cohort 1, patients had stable eGFRs after switching to TAF for 96 weeks, with a median change in eGFR of −0.4 mL/min (interquartile range [IQR], −3.9 to 4.5) at week 24, −0.5 mL/min (IQR, −4.1 to 3.0) at week 48, and 1.0 mL/min (IQR, −2.8 to 4.5) at week 96 [50]. In the subgroup of patients switched from non‐TDF antivirals (mostly ETV) to TAF, the median change in eGFR was negligible [50]. The eGFR changes in cohort 2, however, were not reported [50].

A recent retrospective study included CHB patients with CKD (eGFR < 60 mL/min/1.73 m^2^; *n* = 100) and without CKD (*n* = 314) who were switched to TAF from ≥ 2‐year treatment with ETV, TDF, or an NUC combination (approximately 40%, 30%, and 30%, respectively) [51]. At 5 years, eGFR remained stable in both CKD (prior ETV, −1.8 ± 3.4 mL/min/1.73 m^2^; prior TDF or combo, +0.6 ± 3.3 mL/min/1.73 m^2^) and non‐CKD subgroups (prior ETV, −3.9 ± 4.5 mL/min/1.73 m^2^; prior TDF or combo, −3.0 ± 4.8 mL/min/1.73 m^2^), indicating the long‐term renal safety of TAF [51].

In clinical practice, while TAF is not recommended in patients with creatinine clearance (CrCl) < 15 mL/min not receiving chronic haemodialysis, dose modification of TAF is not required in individuals with renal impairment [14]. In contrast, the dose of ETV should be reduced based on reduced CrCl (Table 3) [14]. Compared with ETV, TAF may offer less variable clinical efficacy using a full dose, while having a neutral effect on renal function, in patients with kidney dysfunction.

**TABLE 3:** Recommended renal dosing of first‐line nucleos(t)ide analogues [14].

Drug	Creatinine clearance (mL/min)			
	≥ 50	30–49	10–29	< 10 with haemodialysis
TAF	25 mg once daily	25 mg once daily	25 mg once daily	25 mg once daily^a^
ETV	0.5 mg once daily	0.25 mg once daily, or 0.5 mg every 48 h	0.15 mg once daily, or 0.5 mg every 72 h	0.05 mg once daily, or 0.5 mg every 7 days
ETV (decompensated liver disease)	1 mg once daily	0.5 mg once daily, or 1 mg every 48 h	0.3 mg once daily, or 1 mg every 72 h	0.1 mg once daily, or 1 mg every 7 days
TDF	300 mg every 24 h	300 mg every 48 h	300 mg every 72–96 h	300 mg every 7 days, or 300 mg after a total of ~12 h of dialysis

Abbreviations: ETV, entecavir; TDF, tenofovir disoproxil fumarate.

^a^ Tenofovir alafenamide (TAF) is not recommended in patients with creatinine clearance < 15 mL/min not receiving chronic haemodialysis.

### 6.2 Kidney Transplant Recipients (KTRs)

KTRs generally require lifelong triple immunosuppression comprising a corticosteroid, a calcineurin inhibitor, and mycophenolate mofetil, and prolonged NUC treatment is often required for patients with CHB. The choice of NUC in KTRs with CHB therefore should not only take into consideration the antiviral potency and resistance but also the impact of NUC on renal allograft function.

Previous studies have reported the short‐ and long‐term efficacy and renal safety of ETV in KTRs [52, 53], but its use was associated with high rates of resistance in patients with prior lamivudine exposure [54, 55].

A recent retrospective study in Hong Kong and Taiwan reported the outcomes of TAF and ETV in KTRs with CHB [56]. The TAF cohort included four treatment‐naïve patients and 35 treatment‐experienced patients (12 of whom received prior ETV), with a mean treatment duration of 26.4 and 43.7 months, respectively; while the ETV cohort included 10 patients who continued the treatment for a median of 5.2 years [56]. The TAF cohort showed a favourable renal allograft survival rate of 89.4%, with a relatively stable mean eGFR throughout the study period (43.6 mL/min at baseline vs. 47.4 mL/min at year 2; *p* = 0.493) [56]. The slopes of eGFR change were comparable before and after TAF treatment (−0.5 ± 6.6 vs. −0.2 ± 7.2 mL/min/year; *p* = 0.937) [56]. Additionally, TAF was not associated with significant changes in kidney injury markers (kidney injury molecule‐1 and interleukin‐18), further supporting the renal safety of TAF in KTRs [56]. There was also no significant difference in eGFR changes between the ETV and TAF cohorts during the study period [56].

### 6.3 Elderly Patients

A retrospective study analysed 246 Japanese patients treated with ETV or TAF for ≥ 2 years into two groups by age: < 65 years (*n* = 130) and ≥ 65 years (*n* = 116) [57]. While both age groups showed significant differences in median eGFR between baseline and 1 or 2 years after antiviral treatment, the older group showed significantly greater eGFR reductions at both year 1 (−2.91 vs. −2.16 mL/min/1.73 m^2^; *p* < 0.001) and year 2 (−4.93 vs. −1.91 mL/min/1.73 m^2^; *p* < 0.001) than the younger group [57].

In patients treated with ETV, the median eGFR significantly decreased from baseline to year 1 or year 2, regardless of age group (< 65 years: −2.27 or −2.47 mL/min/1.73 m^2^; ≥ 65 years: −3.51 or −5.31 mL/min/1.73 m^2^) [57]. In the TAF group, those aged < 65 years had no significant eGFR reductions from baseline to year 1 or year 2 (−1.69 or 0.32 mL/min/1.73 m^2^), whereas those aged ≥ 65 years only had a significant eGFR reduction from baseline to year 2 (−4.11 mL/min/1.73 m^2^; [−1.57 for year 1]) [57]. These findings suggest that, compared with ETV, TAF may be less nephrotoxic in patients aged < 65 years and associated with a later and numerically smaller decline in eGFR in patients aged ≥ 65 years [57]. The small number of patients treated with TAF (62 vs. 184 for ETV), however, is a major limitation [57].

### 6.4 Patients With HBV‐Related Acute‐On‐Chronic Liver Failure (ACLF)

A prospective cohort study included 199 patients with HBV‐ACLF who received ETV, TAF, or TDF for 144 weeks in China [58]. The PSM data on 44 patients in each treatment group showed that, at week 144, there were no significant differences in changes in serum creatinine (ETV, −4.00 μmol/L; TAF, 4.95 μmol/L; TDF, 5.65 μmol/L; *p* = 0.349) and eGFR (figures not reported; *p* = 0.768) between the different NUCs [58].

In a retrospective study comprising 172 ETV‐treated and 100 TAF‐treated patients with HBV‐ACLF in China, PSM data on 100 pairs demonstrated that the TAF group had a significantly greater eGFR increase from baseline to 4 weeks after treatment compared with the ETV group (5.98 ± 14.46 vs. 1.18 ± 18.07 mL/min/1.73 m^2^; *p* < 0.001) [59]. At week 48, TAF was associated with a significantly lower risk of CKD progression in patients with CKD stage 1 at baseline compared with ETV (7.3% [4/55] vs. 34.8% [16/46] had a minimum one‐stage elevation; *p* = 0.001) [59]. The TAF group also had a significantly higher rate of liver transplant‐free survival compared with the ETV group (76% [76/100] vs. 58% [58/100]; *p* = 0.007) [59].

A recent network meta‐analysis included nine prospective or retrospective cohort studies of 939 patients with HBV‐ACLF who received ETV, TAF, or TDF [60]. Based on SUCRA values, TAF ranked lowest for risk of eGFR reduction (13.9% vs. 97.5% for ETV and 38.7% for TDF) and serum creatinine increase (1.2% vs. 77.0% for ETV and 71.9% for TDF) [60]. Compared with ETV, TAF was associated with smaller changes in eGFR (SMD, −0.35; 95% CI, −0.52 to 0.18; *p* value not reported) and serum creatinine (SMD, 0.30; 95% CI, 0.09–0.51; *p* value not reported) from baseline to 4 weeks after treatment [60].

## 7 Discussion

The management of CHB has evolved significantly over the past few decades from the use of first‐generation NUC lamivudine to current standard first‐line treatments with ETV, TDF, and TAF [13, 14, 15]. While HBV suppression and clinical outcomes have improved significantly with the use of NUCs, new challenges have arisen as a result of prolonged drug administration and changes in patient demographics. In this context, the ageing population in many countries/localities faces natural decline in kidney function and development of CKD risk factors such as DM and HT [61]. In 2023, the global age‐adjusted prevalence of CKD was 14.2% among adults aged ≥ 20 years, translating to an estimated 788 million patients with CKD worldwide, a remarkable rise from 378 million in 1990 and 627 million in 2013 [62]. China (152 million) and India (138 million) had the highest numbers of patients with CKD in 2023; countries that had more than 10 million patients with CKD included Bangladesh, Brazil, Indonesia, Iran, Japan, Mexico, Nigeria, Pakistan, the Philippines, Russia, Thailand, Türkiye, the USA, and Vietnam [62]. A predominance of more severe CKD stages was observed in older adults [62]. In Hong Kong, the median age of new patients with kidney failure with replacement therapy in 2022 was 63.4 years, representing a substantial increase from 51.4 years in 2013 [63]. Compared with the general population, patients with CHB have remarkably higher risks of renal abnormalities, eGFR decline, and CKD [3, 4, 5]. Most patients with CHB require prolonged NUC therapy, making renal safety an essential consideration in treatment selection.

The present narrative review closely examined the renal effects of three standard first‐line NUCs in general and special populations with CHB. Although the conventional views have considered ETV to be renally safe, our review of literature suggests that the comparative renal safety of ETV versus TDF remains inconclusive and may vary depending on baseline patient characteristics, such as the presence or absence of pre‐existing renal impairment. Data from one RCT and multiple real‐world studies reported similar renal safety profiles for ETV and TDF, particularly among patients with no or mild pre‐existing kidney dysfunction. However, such conclusions may be limited by the nature of retrospective studies, including selection bias and heterogeneous patient characteristics (e.g., age, comorbidities, treatment duration). Indeed, long‐term prospective studies (with the use of more sensitive kidney biomarkers) are warranted to answer whether ETV or TDF is more nephrotoxic in general and special populations with CHB.

Differences in pharmacological properties have resulted in the lower nephrotoxic potential of TAF compared with TDF, with robust clinical data showing a much favourable renal safety profile for TAF. The comparative nephrotoxicity between TAF and ETV, however, remains controversial. Despite a lack of prospective randomised data, real‐world evidence on renal safety in treatment‐naïve and treatment‐experienced patients with CHB generally appears to favour TAF over ETV. The renal safety of TAF has also been observed in a wide spectrum of special populations with CHB, including patients with pre‐existing moderate‐to‐severe renal impairment, KTRs, elderly patients, and patients with HBV‐ACLF. While TAF offers improved, or at least comparable, renal safety, it may provide several advantages over ETV: (1) a more consistent antiviral effect in patients with renal impairment (as no dose adjustments are needed in most renal clearance states); (2) effective for both treatment‐naïve and NUC‐experienced subjects. Caveats of the current literature comparing ETV and TAF include selection bias linked to retrospective design, a paucity of non‐Asian patients, relatively small sample sizes, and short treatment duration or follow‐up. While many cited studies have reported small absolute changes in eGFR, it remains uncertain whether these numerically minimal declines are clinically meaningful. Indeed, the threshold for eGFR drop for clinical actions (e.g., change of NUC or initiation of reno‐protective medications) is undefined. Moreover, it is also important to interpret the eGFR drop with respect to the clinical context. For instance, in patients with pre‐existing CKD or KTRs, even a numerically small decline in eGFR may translate into more sinister long‐term renal consequences after prolonged NUC treatments.

DM shows a growing prevalence globally and is an important risk factor for CKD. The presence of DM in CHB patients will render them more vulnerable to kidney impairment and CKD [61, 63], but how concomitant DM and HBV infection may interact to accelerate CKD progression remains unclear. Further basic and clinical studies to examine this link and the optimal choice of NUC for this special population have important implications. The early use of an efficacious NUC with a good renal safety profile potentially prevents acute and/or chronic nephrotoxicity in these susceptible individuals.

## 8 Conclusions

Current first‐line NUCs show comparable renal safety profiles in CHB patients with normal kidney function, with growing evidence that favours TAF. Future prospective studies are needed to validate these findings, and more research should focus on CHB patients with DM who are at risk of CKD.

## Author Contributions

**L.Y.M.:** conceptualisation, supervision, writing – review and editing. **T.H.H.:** writing – review and editing, validation, investigation. **D.Y.H.Y.:** project administration, writing – original draft, Writing – review and editing. All authors approved the final version of the article, including the authorship list. Guarantor of article: Desmond Y. H. Yap.

## Funding

Editing of this manuscript, which was provided by Best Solution Co. Ltd., was supported by an independent grant from Gilead Sciences.

## Ethics Statement

The authors have nothing to report.

## Consent

The authors have nothing to report.

## Conflicts of Interest

L.Y.M. received research funding support and speaker fee from Gilead Sciences. The other authors declare no conflicts of interest.

## Data Availability

Data sharing not applicable to this article as no datasets were generated or analysed during the current study.
